# Supplementary material for: Family Support as Smoking Prevention during Transition from Early to Late Adolescence: A Study in 42 Countries
Source: Int J Environ Res Public Health. 2021 Dec 2;18(23):12739. doi: 10.3390/ijerph182312739 (PMC8656923; doi:10.3390/ijerph182312739)
Supplement: Supplementary file 1 [file ijerph-18-12739-s001.zip › ijerph-1449000-supplementary.pdf]

Table S1. Country-level data on smoking prevalence, family support, and the association of these factors

No	Country	V1	V2	V3	V4	V4	V6	V7	V8	V9
1	Albania	1634	2.86	87.60	86.10	88.80	87.10	87.60	84.60	94.20
2	Armenia	3774	1.24	77.80	75.10	80.20	78.40	74.20	72.80	80.70
3	Austria	3845	1.21	74.90	76.90	73.00	83.70	76.10	70.80	85.50
4	Belgium (Flemish)	3863	1.21	73.80	77.80	70.10	82.90	78.90	70.70	80.70
5	Belgium (French)	3514	1.33	73.80	76.90	71.00	80.90	77.50	67.20	75.70
6	Bulgaria	4407	1.06	38.30	35.70	40.90	41.30	34.60	30.00	42.50
7	Canada	11461	.41	48.30	50.80	46.10	58.50	50.50	44.70	55.90
8	Switzerland	6711	.70	75.90	77.70	74.10	81.80	77.00	74.30	78.80
9	Czech Republic	10475	.45	58.70	60.00	57.30	64.20	59.80	56.40	62.70
10	Germany	3795	1.23	72.90	72.50	73.20	77.60	75.00	65.60	83.30
11	Denmark	2995	1.56	82.50	84.70	80.40	87.10	81.70	84.80	86.00
12	Estonia	4483	1.04	75.50	78.20	72.80	84.20	79.30	70.90	82.70
13	Spain	4110	1.14	79.60	80.80	78.40	91.30	81.70	71.80	91.20
14	Finland	2962	1.58	68.80	72.50	65.30	81.50	70.90	66.30	76.70
15	France	7923	.59	72.00	74.40	69.70	78.70	74.00	68.90	78.10
16	England	2763	1.69	53.10	56.40	49.50	66.00	50.60	49.70	59.70
17	Scotland	4589	1.02	59.50	59.30	59.70	72.40	58.10	44.50	73.00
18	Wales	12009	.39	61.40	64.00	59.00	72.90	64.60	54.20	72.70
19	Georgia	3685	1.27	66.10	65.80	66.40	69.50	68.40	58.90	73.60
20	Greece	3631	1.29	77.00	80.70	73.50	86.10	81.40	74.90	84.50
21	Croatia	4508	1.04	79.10	80.30	78.00	86.60	80.20	75.90	91.10
22	Hungary	3538	1.32	82.20	84.70	80.10	92.20	83.90	77.40	88.90
23	Ireland	3526	1.32	61.00	61.60	60.40	68.50	59.90	55.20	69.00
24	Iceland	6530	.71	71.10	71.30	70.90	79.00	72.00	62.40	81.00
25	Italy	3914	1.19	75.70	78.00	73.60	81.40	80.10	71.40	80.80
26	Kazakhstan	4208	1.11	80.80	79.00	82.50	82.30	79.50	74.30	84.30
27	Lithuania	3578	1.30	72.20	74.30	70.00	82.20	72.90	66.70	80.30
28	Luxembourg	3712	1.26	71.60	74.80	68.50	84.10	77.30	66.20	76.90
29	Latvia	4118	1.13	66.20	67.70	64.70	73.70	66.90	62.30	76.90
30	Moldova	4344	1.07	75.30	73.80	76.70	76.80	76.90	67.50	80.70
31	North Macedonia	4251	1.10	89.70	89.90	89.50	90.60	91.00	88.00	94.00
32	Malta	2252	2.07	73.10	71.10	74.90	82.20	72.20	49.00	87.10
33	Netherlands	4444	1.05	81.20	81.70	80.70	85.20	79.60	81.00	86.80
34	Poland	4895	.95	59.80	61.80	57.90	78.80	58.20	48.40	75.30
35	Portugal	5493	.85	79.20	81.00	77.60	88.90	80.70	74.00	87.90
36	Romania	4283	1.09	80.80	83.30	78.50	85.90	82.60	81.90	85.00
37	Serbia	3541	1.32	84.50	83.40	85.50	92.00	83.00	79.20	94.10
38	Russia	3907	1.19	65.00	65.80	64.30	75.80	68.80	57.30	74.50
39	Sweden	3842	1.21	79.20	81.70	76.70	86.50	81.70	78.00	83.30
40	Slovenia	5279	.88	61.10	61.80	60.50	68.70	62.20	53.80	67.70
41	Slovakia	3121	1.49	70.10	71.40	68.90	81.40	71.70	61.30	82.10
42	Ukraine	6053	.77	57.20	56.40	58.00	64.70	55.70	48.80	62.60
43	TOTAL	195966		71.28	72.41	70.19	78.90	72.36	65.76	78.77

Table S1. (Continuation 1)

No	V10	V11	V12	V13	V14	V15	V16	V17	V18	V19	V20
1	92.20	82.40	10.40	3.80	2.70	6.30	17.50	1.10	3.30	6.40	14.80
2	80.60	79.40	8.40	.90	2.70	7.30	15.60	.30	1.00	1.20	12.90
3	72.00	63.60	6.10	7.40	.90	3.00	15.60	.50	2.80	17.70	14.70
4	69.70	57.90	4.70	3.70	.30	2.10	12.20	.50	2.00	8.90	11.90
5	67.60	62.30	5.20	4.10	1.60	4.90	12.60	.70	3.40	12.80	11.00
6	35.80	43.20	15.00	19.50	8.10	12.40	26.00	8.80	10.60	37.70	17.90
7	46.10	38.40	4.90	5.20	1.70	3.10	9.20	1.00	3.90	9.60	7.50
8	74.50	68.70	6.90	5.70	1.00	3.80	16.50	.20	3.10	14.40	15.50
9	58.40	51.00	7.50	9.10	1.50	5.20	15.60	.40	6.50	20.60	14.10
10	73.70	65.50	6.50	7.50	.00	4.70	14.60	.60	4.80	15.00	14.60
11	75.40	77.10	6.10	4.60	.80	4.20	17.70	.40	2.40	15.80	16.90
12	68.60	67.00	9.20	8.90	1.60	7.20	19.20	1.40	8.90	16.60	17.60
13	76.10	70.70	6.50	7.80	.40	2.90	14.90	.30	4.80	17.00	14.50
14	63.00	57.40	9.60	8.20	1.30	7.70	19.00	.60	6.50	16.50	17.70
15	67.40	62.40	6.50	6.70	2.00	5.10	15.20	.70	4.70	17.50	13.20
16	45.30	39.70	4.30	5.60	.80	3.60	11.10	.90	4.90	13.00	10.30
17	55.90	47.20	4.20	4.50	.10	3.20	10.40	.50	3.40	10.20	10.30
18	55.50	49.40	5.20	6.50	1.50	4.40	10.70	1.00	5.30	15.60	9.20
19	63.30	62.30	7.10	3.30	2.60	4.00	15.60	3.40	2.10	4.40	13.00
20	70.90	65.40	7.50	8.30	1.00	4.70	15.90	.50	5.20	18.80	14.90
21	77.80	68.80	12.40	11.90	1.50	6.20	25.20	.50	6.30	24.10	23.70
22	80.90	70.60	8.10	11.00	2.10	4.90	20.60	1.90	5.80	25.40	18.50
23	60.50	51.50	3.90	3.60	.30	3.00	9.60	.00	1.90	9.90	9.30
24	70.10	61.60	3.70	2.30	2.10	2.50	7.00	.40	1.10	5.50	4.90
25	73.80	65.90	9.30	13.60	1.50	5.00	23.80	.40	7.10	32.90	22.30
26	83.50	79.70	3.70	2.40	1.90	2.70	6.80	1.90	1.90	3.50	4.90
27	65.00	63.70	15.30	13.90	5.10	12.60	30.60	3.20	12.10	27.50	25.50
28	71.10	60.30	8.10	8.10	.60	3.70	12.10	1.80	4.90	14.40	11.50
29	63.50	52.10	10.00	10.40	3.60	7.00	20.70	.90	8.00	24.00	17.10
30	78.80	70.90	6.90	2.10	2.00	4.50	14.30	.80	1.00	4.60	12.30
31	89.50	84.70	7.90	5.00	2.00	3.30	19.20	.80	2.30	12.40	17.20
32	68.60	63.10	4.30	3.80	.00	1.60	14.00	.40	4.50	7.60	14.00
33	83.00	72.50	5.80	4.70	.60	3.70	13.20	.10	1.40	12.60	12.60
34	55.10	45.00	8.40	9.50	3.00	6.00	16.40	1.20	6.80	19.50	13.40
35	75.50	71.00	5.50	4.70	1.30	3.80	11.80	1.00	3.20	9.40	10.50
36	76.60	74.70	11.20	10.80	2.60	7.70	22.90	1.70	7.70	22.00	20.30
37	86.00	79.30	9.10	9.90	2.00	3.50	17.70	.90	2.40	21.90	15.70
38	67.00	56.40	7.50	5.10	2.90	5.40	12.20	1.30	3.80	8.40	9.30
39	77.00	71.80	5.70	6.50	1.60	3.80	10.60	.20	4.50	12.30	9.00
40	62.60	48.80	6.50	5.90	1.90	4.50	13.80	.20	2.90	16.50	11.90
41	66.70	57.60	10.20	9.70	2.90	9.00	20.20	1.50	9.10	22.10	17.30
42	57.10	53.40	9.20	4.80	3.00	5.90	19.00	.90	4.40	10.10	16.00
43	69.09	62.72	7.49	6.93	1.84	5.00	15.88	1.09	4.59	15.15	14.04

Table S1. (Continuation 2)

No	V21	V22	V23	V24	V25	V26	V27	V28	V29	V30	V31
1	5.30	.000	2.694	1.718	4.225	.019	2.015	1.123	3.615	.000	4.305
2	.90	.000	3.148	2.231	4.441	.000	2.680	1.858	3.865	.007	4.134
3	17.20	.000	3.214	2.476	4.172	.000	2.731	1.836	4.061	.000	3.617
4	8.40	.000	3.096	2.232	4.296	.000	2.562	1.646	3.988	.000	4.353
5	12.10	.000	2.466	1.754	3.465	.000	2.937	1.820	4.740	.002	2.129
6	28.90	.000	1.447	1.233	1.699	.000	1.867	1.426	2.444	.023	1.266
7	8.60	.000	2.925	2.310	3.704	.000	2.560	1.849	3.544	.000	3.391
8	14.20	.000	2.495	2.042	3.049	.000	1.912	1.443	2.532	.000	3.481
9	20.20	.000	1.813	1.577	2.084	.000	1.698	1.381	2.087	.000	1.896
10	14.40	.000	1.906	1.494	2.431	.263	1.246	.848	1.830	.000	2.610
11	15.40	.000	1.994	1.402	2.837	.097	1.555	.923	2.620	.000	2.671
12	15.20	.000	2.102	1.709	2.586	.000	1.916	1.421	2.584	.000	2.323
13	16.70	.000	2.607	2.049	3.317	.000	2.461	1.702	3.559	.000	2.718
14	15.90	.000	2.802	2.163	3.630	.000	2.529	1.772	3.610	.000	3.277
15	16.80	.000	2.213	1.844	2.655	.000	2.085	1.588	2.736	.000	2.300
16	12.10	.000	2.463	1.707	3.553	.012	1.977	1.163	3.359	.000	2.881
17	9.70	.000	3.090	2.257	4.231	.000	3.726	2.298	6.044	.000	2.665
18	14.60	.000	2.440	2.059	2.892	.000	2.178	1.675	2.832	.000	2.584
19	1.00	.000	1.726	1.298	2.295	.000	1.872	1.319	2.657	.139	1.450
20	18.30	.000	2.121	1.654	2.721	.004	1.786	1.205	2.648	.000	2.333
21	23.60	.000	2.579	2.154	3.089	.000	2.266	1.742	2.947	.000	2.943
22	23.50	.000	2.565	1.968	3.343	.003	2.089	1.275	3.424	.000	2.647
23	9.90	.000	2.834	1.935	4.149	.000	3.127	1.846	5.296	.001	2.523
24	5.10	.000	3.969	2.906	5.419	.000	3.970	2.647	5.956	.000	3.909
25	32.50	.000	2.343	1.929	2.846	.000	2.076	1.496	2.880	.000	2.427
26	1.60	.000	2.996	2.092	4.289	.000	2.314	1.450	3.692	.000	4.208
27	24.30	.000	1.700	1.418	2.038	.002	1.499	1.158	1.940	.000	1.956
28	12.60	.000	2.267	1.786	2.876	.000	2.331	1.660	3.273	.000	2.223
29	23.10	.000	2.653	2.167	3.247	.000	2.210	1.661	2.940	.000	3.201
30	3.80	.000	2.588	1.933	3.463	.000	2.443	1.752	3.407	.001	2.693
31	11.60	.000	3.296	2.479	4.382	.000	2.458	1.645	3.671	.000	4.715
32	7.20	.000	4.028	2.521	6.436	.000	4.852	2.254	10.447	.000	3.541
33	12.50	.000	2.170	1.647	2.860	.002	1.840	1.247	2.714	.000	2.638
34	18.30	.000	2.443	2.000	2.983	.000	2.058	1.533	2.763	.000	2.828
35	8.40	.000	3.241	2.550	4.121	.000	2.793	1.994	3.912	.000	3.871
36	20.30	.000	2.315	1.881	2.850	.000	1.814	1.318	2.496	.000	2.842
37	21.00	.000	2.934	2.322	3.707	.000	2.806	1.989	3.958	.000	2.981
38	7.10	.000	2.541	1.934	3.339	.000	2.673	1.874	3.813	.000	2.415
39	12.10	.000	3.092	2.350	4.069	.000	3.109	2.063	4.683	.000	3.064
40	16.30	.000	3.038	2.392	3.858	.000	2.534	1.831	3.507	.000	3.727
41	20.60	.000	2.593	2.053	3.275	.000	2.473	1.769	3.456	.000	2.697
42	9.20	.012	1.378	1.072	1.771	.000	1.819	1.398	2.367	.000	4.011
43	14.06	.000	1.991	1.918	2.067	.000	1.807	1.714	1.905	.000	2.194

Table S1. (Continuation 3)

No	V32	V33	V34	V35	V36	V37	V38	V39	V40	V41	V42
1	2.074	8.933	.062	5.747	.916	36.057	.784	1.383	.136	14.028	.157
2	1.470	11.628	.046	4.645	1.027	20.998	.000	3.782	1.937	7.386	.005
3	2.548	5.133	.779	1.393	.138	14.049	.000	6.953	2.402	20.129	.017
4	2.603	7.281					.089	3.004	.846	10.667	.031
5	1.312	3.456	.008	5.087	1.544	16.765	.117	2.175	.823	5.747	.058
6	1.034	1.550	.146	1.514	.865	2.649	.001	3.025	1.591	5.749	.038
7	2.395	4.802	.013	5.168	1.417	18.853	.004	2.642	1.352	5.162	.001
8	2.588	4.683	.014	4.159	1.337	12.936	.002	2.768	1.458	5.255	.040
9	1.568	2.291	.006	3.346	1.416	7.905	.059	1.517	.985	2.336	.001
10	1.891	3.603					.756	1.132	.518	2.471	.951
11	1.631	4.374				.000	.002	4.296	1.736	10.634	.943
12	1.734	3.111	.652	1.428	.304	6.719	.001	2.744	1.547	4.867	.203
13	1.974	3.742				.000	.000	5.487	2.254	13.358	.187
14	2.225	4.826	.802	1.340	.136	13.166	.175	1.575	.817	3.038	.000
15	1.795	2.947	.000	6.731	2.667	16.989	.186	1.411	.847	2.349	.005
16	1.717	4.835	.963	1.068	.067	17.095	.595	.765	.284	2.056	.009
17	1.760	4.036					.006	4.154	1.499	11.512	.018
18	2.064	3.234	.087	2.439	.878	6.774	.001	2.277	1.409	3.680	.035
19	.886	2.374	.641	.763	.245	2.376	.046	2.168	1.013	4.637	.018
20	1.680	3.240					.067	2.128	.948	4.776	.170
21	2.292	3.780	.090	4.511	.790	25.747	.000	3.430	1.757	6.696	.001
22	1.922	3.644	.654	1.628	.194	13.696	.010	3.579	1.348	9.502	.492
23	1.452	4.386	.619	2.023	.126	32.490	.067	2.564	.937	7.020	.005
24	2.397	6.375	.001	4.187	1.735	10.105	.000	6.676	2.604	17.115	.000
25	1.899	3.101	.396	1.840	.450	7.525	.054	2.225	.987	5.015	.025
26	2.381	7.439	.205	2.036	.678	6.114	.014	3.056	1.254	7.446	.174
27	1.511	2.533	.434	1.405	.599	3.296	.515	1.179	.719	1.932	.153
28	1.588	3.113	.332	3.456	.282	42.291	.200	1.863	.720	4.822	.001
29	2.395	4.279	.004	3.440	1.490	7.942	.062	1.726	.973	3.063	.000
30	1.470	4.932	.303	1.786	.592	5.390	.000	4.351	2.101	9.012	.010
31	3.115	7.137	.002	7.627	2.134	27.261	.400	1.659	.511	5.384	.002
32	1.935	6.478					.302	3.642	.313	42.421	.109
33	1.780	3.911	.115	4.963	.676	36.441	.001	3.892	1.795	8.442	.537
34	2.146	3.727	.008	3.135	1.343	7.320	.724	1.114	.613	2.024	.020
35	2.739	5.472	.590	1.530	.326	7.187	.000	3.271	1.701	6.289	.018
36	2.146	3.764	.004	4.551	1.628	12.722	.036	2.064	1.049	4.062	.224
37	2.164	4.106	.559	1.896	.222	16.182	.000	10.679	3.536	32.250	.002
38	1.575	3.703	.012	3.929	1.353	11.412	.001	3.221	1.573	6.594	.006
39	2.113	4.442					.033	2.598	1.082	6.236	.000
40	2.606	5.329	.478	1.522	.477	4.853	.000	4.789	2.148	10.674	.004
41	1.945	3.741	.031	4.527	1.150	17.815	.006	2.172	1.256	3.758	.012
42	2.739	5.874	.006	3.577	1.443	8.867	.006	2.201	1.249	3.877	.232
43	2.081	2.314	.000	2.930	2.427	3.536	.000	2.385	2.137	2.663	.000

Table S1. (Continuation 5)

No	V43	V44	V45	V46	V47	V48	V49	V50	V51	V52	V53
1	1.606	.833	3.097	.006	28.964	2.626	319.472	.411	2.518	.278	22.807
2	1.989	1.235	3.203					.008	9.345	1.800	48.500
3	1.751	1.107	2.771					.001	5.858	2.052	16.722
4	1.697	1.049	2.744	.061	8.922	.907	87.784	.086	3.193	.849	12.001
5	1.875	.979	3.591	.677	1.444	.257	8.120	.955	1.034	.332	3.218
6	1.456	1.021	2.076	.004	2.218	1.282	3.839	.079	1.713	.939	3.128
7	1.945	1.304	2.901					.003	2.648	1.383	5.067
8	1.411	1.016	1.960	.099	7.765	.678	88.920	.000	3.475	1.729	6.986
9	1.488	1.165	1.901	.302	2.201	.491	9.858	.001	1.935	1.308	2.863
10	1.014	.649	1.584	.114	4.864	.684	34.582	.000	4.434	2.002	9.822
11	.974	.470	2.017					.000	10.966	2.959	40.633
12	1.267	.880	1.823	.954	1.047	.216	5.076	.000	3.149	1.862	5.327
13	1.321	.874	1.999	.210	5.889	.368	94.149	.002	2.934	1.499	5.741
14	2.329	1.503	3.608					.000	4.358	1.941	9.782
15	1.665	1.169	2.372	.542	1.753	.288	10.662	.000	2.969	1.856	4.750
16	2.469	1.259	4.842	.125	5.561	.621	49.837	.283	1.682	.651	4.349
17	1.954	1.121	3.404	.050	9.242	1.003	85.124	.297	1.521	.692	3.342
18	1.436	1.026	2.009	.147	2.663	.708	10.021	.000	3.314	2.058	5.337
19	1.690	1.095	2.608	.748	1.158	.473	2.836	.660	1.269	.439	3.665
20	1.375	.872	2.167	.384	2.916	.262	32.462	.075	1.852	.939	3.651
21	1.648	1.230	2.209	.144	6.019	.543	66.764	.000	3.480	1.801	6.724
22	1.238	.674	2.276					.001	3.342	1.610	6.938
23	2.525	1.329	4.797					.031	4.320	1.142	16.351
24	2.575	1.516	4.373	.045	10.254	1.051	100.037	.002	11.071	2.358	51.973
25	1.532	1.054	2.226	.313	4.186	.259	67.753	.000	3.351	1.853	6.058
26	1.558	.822	2.955	.000	9.753	2.815	33.790	.338	1.776	.549	5.751
27	1.270	.915	1.764	.609	.726	.213	2.477	.047	1.679	1.007	2.800
28	2.349	1.424	3.876	.037	4.656	1.101	19.696	.150	1.752	.816	3.761
29	1.902	1.330	2.720	.053	4.644	.981	21.981	.000	3.052	1.724	5.403
30	1.692	1.133	2.528	.012	16.513	1.835	148.631	.774	.728	.084	6.319
31	2.041	1.297	3.211					.001	6.848	2.124	22.083
32	1.953	.862	4.429	.137	8.196	.511	131.436	.061	2.656	.957	7.369
33	1.167	.715	1.905					.204	2.390	.623	9.167
34	1.567	1.075	2.285	.218	2.300	.611	8.662	.000	3.698	2.046	6.683
35	1.820	1.106	2.995	.002	7.540	2.113	26.900	.000	3.768	1.895	7.494
36	1.288	.857	1.935	.444	1.851	.382	8.969	.000	4.290	2.415	7.622
37	1.842	1.259	2.697	.006	17.077	2.274	128.244	.116	3.292	.745	14.546
38	1.874	1.202	2.923	.833	.783	.080	7.678	.011	3.219	1.313	7.891
39	3.495	2.148	5.687					.001	4.106	1.841	9.155
40	1.744	1.192	2.553					.035	2.352	1.063	5.200
41	1.760	1.130	2.742	.887	1.174	.130	10.588	.001	2.527	1.489	4.289
42	1.213	.884	1.664	.019	12.432	1.525	101.350	.000	3.887	2.005	7.537
43	1.568	1.472	1.671	.000	3.988	3.168	5.020	.000	3.158	2.815	3.542

Table S1. (Continuation 6)

No	V54	V55	V56	V57
1	.016	2.901	1.222	6.887
2	.226	2.594	.554	12.143
3	.000	2.144	1.463	3.143
4	.001	2.656	1.482	4.762
5	.012	2.092	1.174	3.728
6	.195	1.170	.923	1.484
7	.000	2.547	1.678	3.866
8	.000	2.678	1.922	3.733
9	.000	1.491	1.196	1.857
10	.012	1.599	1.107	2.310
11	.110	1.604	.898	2.865
12	.050	1.462	1.001	2.135
13	.006	1.671	1.156	2.415
14	.004	1.933	1.233	3.032
15	.007	1.506	1.116	2.032
16	.028	2.069	1.080	3.964
17	.010	1.996	1.176	3.387
18	.000	1.627	1.251	2.116
19	.081	1.942	.922	4.090
20	.007	1.696	1.159	2.482
21	.000	1.781	1.353	2.343
22	.004	1.707	1.186	2.459
23	.157	1.557	.843	2.874
24	.007	2.110	1.224	3.638
25	.001	1.600	1.217	2.104
26	.000	4.821	2.098	11.079
27	.001	1.709	1.239	2.356
28	.033	1.601	1.038	2.468
29	.000	1.962	1.391	2.768
30	.065	1.951	.960	3.963
31	.000	2.864	1.832	4.479
32	.109	1.901	.866	4.175
33	.008	1.766	1.163	2.681
34	.007	1.558	1.127	2.153
35	.000	2.510	1.510	4.171
36	.000	1.988	1.410	2.805
37	.000	1.907	1.371	2.652
38	.012	1.915	1.154	3.178
39	.000	2.331	1.526	3.560
40	.000	2.652	1.770	3.974
41	.008	1.792	1.166	2.753
42	.000	3.122	1.931	5.050
43	.000	1.886	1.773	2.007

Note: List of variables and their labels

Variable	Label
V1	Number of subjects in the country
V2	Coefficient for weigthing by countries
V3	Percentage of hight family support in total sample
V4	Percentage of high family suport in boys, 11, 13 & 15 years
V5	Percentage of high family suport in gils, 11, 13 & 15 years
V6	Percentage of high family support in boys, 11 years
V7	Percentage of high family support in boys, 13 years
V8	Percentage of high family support in boys, 15 years
V9	Percentage of high family support in girls, 11 years
V10	Percentage of high family support in girls, 13 years
V11	Percentage of high family support in girls. 15 years
V12	Smoking prevalence, all ages, boys
V13	Smoking prevalence, all ages, girls
V14	Smoking prevalence, 11 boys
V15	Smoking prevalence, 13 boys
V16	Smoking prevalence, 15 boys
V17	Smoking prevalence, 11 girls
V18	Smoking, prevalence, 13 girls
V19	Smoking, prevalence, 15 girls
V20	Smoking prevalence, difference 15-11 boys
V21	Smoking prevalence, difference 15--11 girls
V22	Prevalence ratio (PR), total sample, p-value
V23	PR, total sample, PR-value
V24	PR, total sample, 95% CI min-value
V25	PR, Total sample, 95% CI max-value
V26	PR, boys, p-value
V27	PR, boys, PR-value
V28	PR, boys, 95% CI min-value
V29	PR, boys, 95% CI max-value
V30	PR, girls, p-value
V31	PR, girls, PR-value
V32	PR, girls, 95% CI min-value
V33	PR, girls, 95% CI max-value
V34	PR, boys, 11 years, p-value
V35	PR, boys, 11 years, PR-value
V36	PR, boys, 11 years, 95% CI min-value
V37	PR, boys, 11 years, 95% CI max-value
V38	PR, boys, 13 years, p-value
V39	PR, boys, 13 years, PR-value
V40	PR, boys, 13 years, 95% CI min-value
V41	PR, boys, 13 years, 95% CI max-value
V42	PR, boys, 15 years, p-value
V43	PR, boys, 15 years, PR-value

Variable	Label
V44	PR, boys, 15 years, 95% CI min-value
V45	PR, boys, 15 years, 95% CI max-value
V46	PR, girls, 11 years, p-value
V47	PR, girls, 11 years, PR-value
V48	PR, girls, 11 years, 95% CI min
V49	PR, girls, 11 years, 95% CI max
V50	PR, girls, 13 years, p-value
V51	PR, girls, 13 years, PR-value
V52	PR, girls, 13 years, 95% CI min-value
V53	PR, girls, 13 years, 95% CI max-value
V54	PR, girls, 15 years, p-value
V55	PR, girls, 15 years, PR-value
V56	PR, girls, 15 years, 95% CI min-value
V57	PR, girls, 15 years, 95% CI max-value-value
